# Supplementary material for: Symmetry control of nanorod superlattice driven by a governing force
Source: Nat Commun. 2017 Nov 10;8:1410. doi: 10.1038/s41467-017-01111-4 (PMC5680336; doi:10.1038/s41467-017-01111-4)
Supplement: Supplementary file 1 — Supplementary Information [file 41467_2017_1111_MOESM1_ESM.pdf]

## Supplementary Notes

### Supplementary Note 1. Determination of lattice parameters.

Tetragonal and hexagonal superlattices were imaged by SEM (Supplementary Figs 1**a**, **c**). Their corresponding FFTs were generated using ImageJ (Supplementary Figs 1**b**, **d**). The angle of superlattice is determined by the central angle ($\theta$). The lattice constant of superlattice is obtained by the distances between the center dot and other bright dots in the FFT image. The formula to calculate the distance in real-space is[1]:

$$Rd = \lambda L \tag{1}$$

Here, $R$ is the distance in the reciprocal space, $d$ is the distance in real space, $\lambda$ is the wave length, $L$ is the camera length.

### Supplementary Note 2. Electrostatic potential between two parallel nanorods.

Electrostatic potential between two parallel cylindricalnanorodswith separation $x$ can be given by[2]:

$$U_{\mathrm{ele}}(x) = \frac{2\sqrt{\pi R}l}{\varepsilon_r \varepsilon_0 \kappa^{3/2}} \sigma^2 Li_{1/2}(e^{-\kappa x}) \tag{2}$$

Where $R$ is the radius of the nanorod including the thickness of coated CTAB bilayer, $l$ is the length of the rods, $\varepsilon_0$ is the vacuum permittivity, $\varepsilon_{\mathrm{r}}$ is the relative dielectric constant of solvent, $\sigma$ is the surface charge density, $\kappa$ is Debye length, $Li_{1/2}(z)$ is the polylogarithm function, defined by $Li_{1/2}(\mathrm{z}) = \Sigma_i(z^{\mathrm{i}}/i^{1/2})$.

### Supplementary Note 3. Van der Waals potential between two parallel nanorods.

Van der Waals potential between the two parallel nanorods with separation $x$ can be expressed[3]:

$$U_{van}(x) \approx -\frac{A_{\mathrm{eff}}l}{24R}\left(\frac{R}{x}\right)^{3/2} \tag{3}$$

where $A_{\mathrm{eff}}$ is effective Hamaker constant of nanorods, $R$ is the radius of the nanorod, $l$ is the length of nanorod. For two identical nanorods interacting across medium (water in our work), $A_{\mathrm{eff}}$ could be deduced by the expression:

$$A_{eff} \approx \frac{3}{4}kT\left(\frac{\varepsilon-\varepsilon_{H_2O}}{\varepsilon+\varepsilon_{H_2O}}\right)^2 + \frac{3h\nu_e}{16\sqrt{2}}\frac{\left(n^2-n_{H_2O}^2\right)^2}{\left(n^2+n_{H_2O}^2\right)^{3/2}} \quad (4)$$

where $\varepsilon$ is dielectric constant, $n$ is refractive index, $k$ is *Boltzmann* constant, $T$ is room temperature, $h$ is Planck constant, $\nu_e$ is absorption frequency.

**Supplementary Note 4. Depletion potential between two parallel cylindrical nanorods.**

Based on Asakura and Oosawa's theory[4], the depletion potential is equal to the osmotic pressure ($P_0$) times the overlap volume $V_{ov}$ ($r$). For two parallel cylindrical nanorods with the inter-rod distance $r$,

$$V_{ov}(r) = \frac{1}{2}l\left[(2R_d)^2 \arccos\left(\frac{r}{2R_d}\right) - r\sqrt{(2R_d)^2 - r^2}\right] \quad 2R \le r < 2R_d \quad (5)$$

where $l$ is the effective length of the rods including the thickness of coated CTAB bilayer, $R_d = R + \sigma/2$, $R$ is the radius of the rods including the thickness of coated CTAB bilayer, $\sigma$ is the effective diameter of the CTAB micelles.

The depletion potential is:

$$U_{dep}(r) = -P_0\mathbf{V}_{ov}(r)$$

$$= -\frac{1}{2}lP_0\left[(2R_d)^2 \arccos\left(\frac{r}{2R_d}\right) - r\sqrt{(2R_d)^2 - r^2}\right] \quad 2R \le r < 2R_d \quad (6)$$

where the osmotic pressure induced by the micelles $P_0 = n_{micelles}R_0T$, $n_{micelles}$ is the concentration of CTAB micelles, $R_0$ is the universal gas constant, $T$ is room temperature.

**Supplementary Note 5. Density functional theory (DFT) calculation.**

Adsorption energy for R6G on rod surface and the interaction energy between R6G molecules in R6G chains were calculated using DFT provided by the DMol3 code[5].

In DMol3, the electronic wave function was expanded in a localized atom-centered basis set with each basis function defined numerically on a dense radial grid. We used the double-numeric polarized

(DND) basis sets[6, 7]. The Perdew and Wang parameterization of the local exchange-correlation energy were applied in the local spin density approximation (LSDA) to describe exchange and correlation[8]. The DFT semi local pseudo-potential (DSPP) specifically developed for DMol3 calculations were adopted for the representation of valence configurations ($5s^2 5p^6 5d^{10} 6s^1$ for Au, $4s^2 4p^6 4d^{10} 5s^1$ for Ag, and $4s^2 4p^6 4d^{10}$ for Pd). Each basis function was restricted to within a cutoff radius of $R_{cut}$ = 5.5 Å. Spin-restricted wave functions were employed. A self-consistent field procedure was done with a convergence criterion of $10^{-5}$ a.u. on the energy and electron density.

To evaluate the interaction of R6G molecules with the different surfaces (Au {110}, Ag {110}, Pd{110}, Au{100}, Ag{100}, and Pd{100}), we design the model system. The model system comprises five metal layers, and R6G adsorbates. First we have optimized the lattice parameters for Au, Ag, and Pd. The results are nearly equal to their experimental values, which mean our DFT methods are suitable for our system. The relaxed unit cell was used to construct the surface models. Then we optimized R6G molecules adsorbed on the different surfaces. During the structural optimizations, we allow the adsorbates to move until all forces vanished within $1.0\times10^{-3}$ hartrees per bohr, and fix the metal atoms in the layers in their bulk configurations. The binding energy of adsorbate with the surface is defined as $E_b$ = $E_{total}$(surface+adsorbate) - $E_{total}$(surface) - $E_{total}$(adsorbate).

**Supplementary Note 6. Finite-Difference Time-Domain (FDTD) simulation of field enhancement of the nanorod superlattices.**

The FDTD simulation adopts a periodic boundary condition *(a* = *b* = 21.4 nm for Au nanorod tetragonal and hexagonal superlattices, *a* = *b* = 26.4 nm for Ag nanorod tetragonal and hexagonal superlattices, *a* = *b* = 28.4 nm for Pd nanorod tetragonal and hexagonal superlattices), and field intensity refers to one at position of 8 nm above the surface of single layer superlattices. Here, the nanorod superlattices are illuminated vertically by circularly polarized light from top to bottom. For the three

lasers usually equipped by Raman spectrophotometer, intensities of all tetragonal superlattices (Au, Ag, Pd) for a certain laser wavelength are larger than those of the hexagonal superlattices.

**Supplementary Figures**

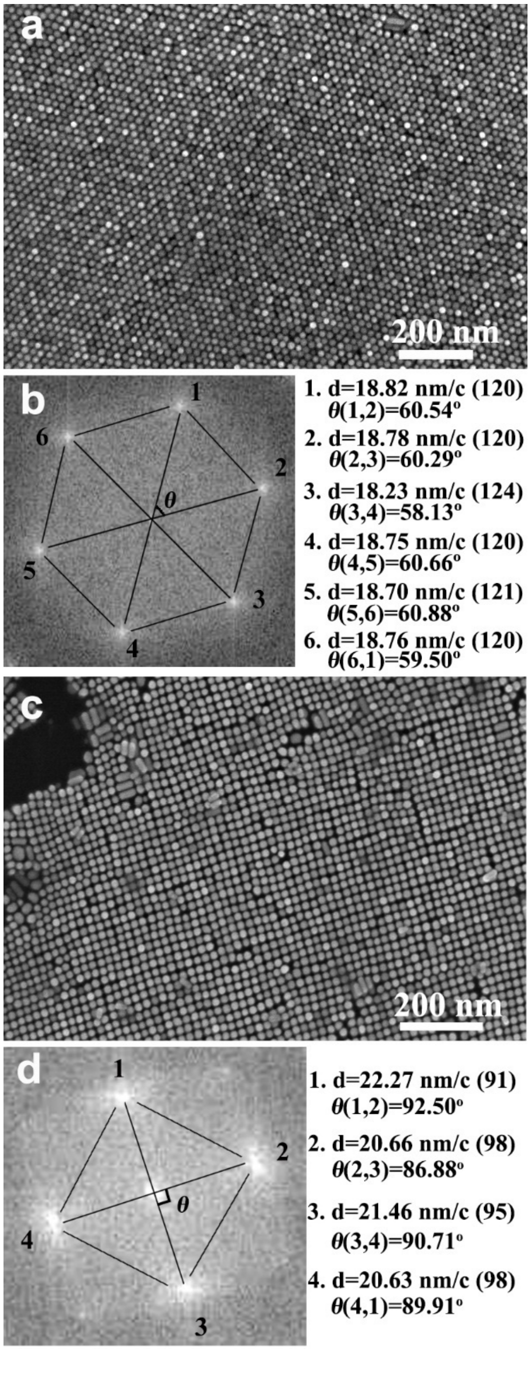


**Supplementary Figure 1. Determination of lattice parameters from SEM.** (**a-b**) SEM image and the corresponding FFT of the hexagonal superlattice. (**c-d**) SEM image and the corresponding FFT of the tetragonal superlattice.

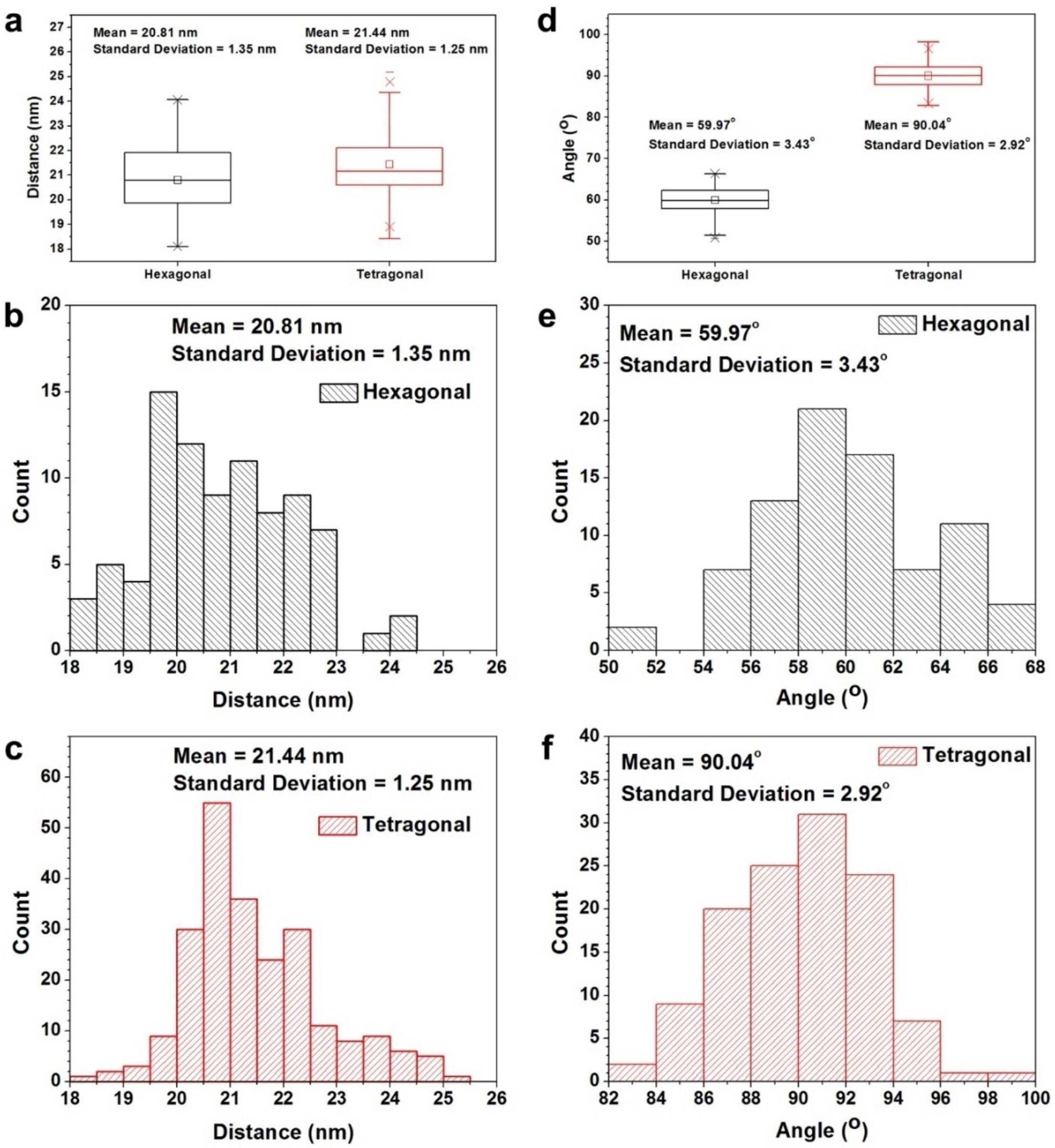


**Supplementary Figure 2. Statistical analyses of lattice parameters.** (**a**) Boxplot of lattice constants of hexagonal and tetragonal superlattices. (**b-c**) Histogram of lattice constants of hexagonal and tetragonal superlattices. (**d**) Boxplot of angles of hexagonal and tetragonal superlattices. (**e-f**) Histogram of angles of hexagonal and tetragonal superlattices. In (**a**) and (**d**), the horizontal bars represent the maximum (top bar) and minimum (bottom bar) values. The crosses demonstrate the 99th percentile (top cross) and 1st percentile (bottom cross). The upper and lower borders of the box are the upper and lower quartiles, respectively. The horizontal line in the box is the median. The little square in box is the mean value.

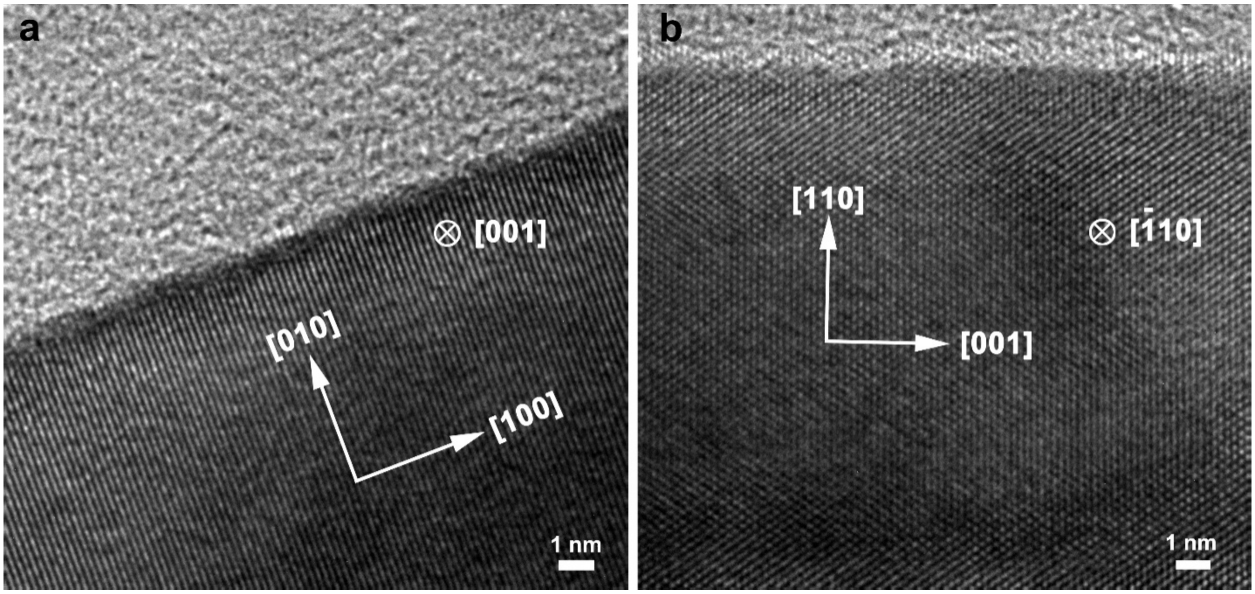


**Supplementary Figure 3. HRTEM images of PC GNRs.** The side facets are composed of two sets of {100} and {110}. Electron beam is aligned on [001] direction (**a**) and [-110] direction (**b**), respectively.

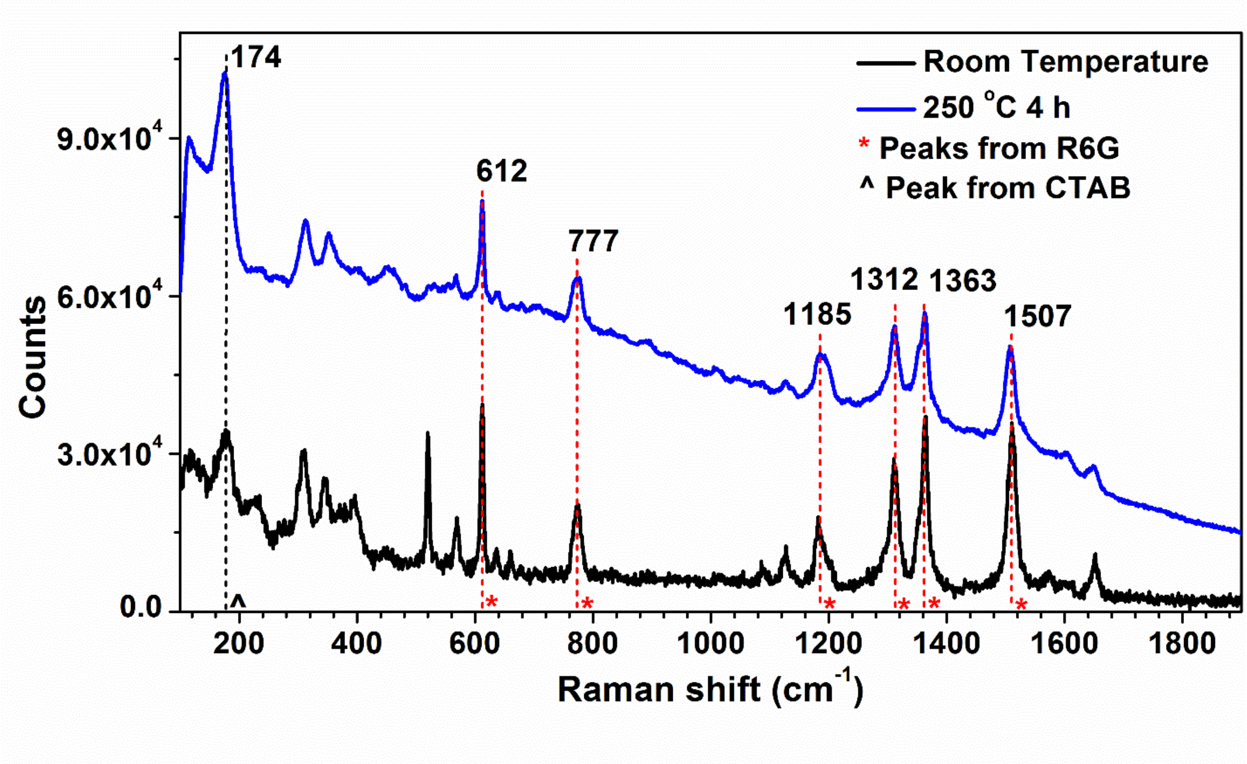


**Supplementary Figure 4. Room temperature SERS spectra of tetragonal superlattice.** Black: as-prepared sample. Blue: sample is heated in air for 4 h at 250 °C and cooled down to the room temperature.

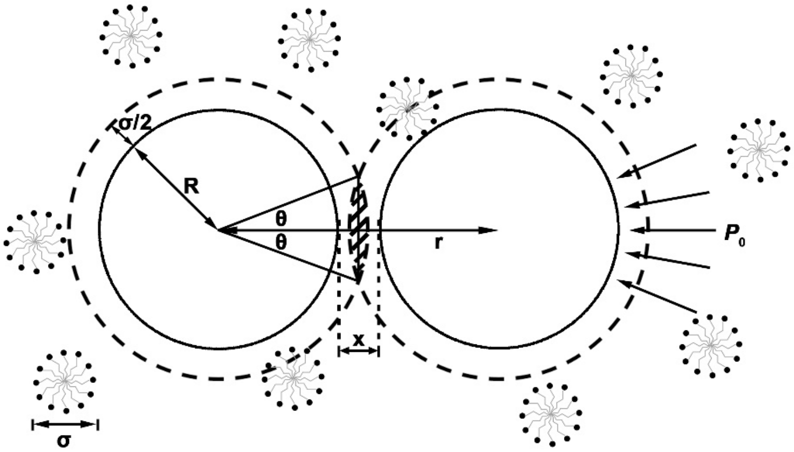


**Supplementary Figure 5. Top view of model for depletion potential between two parallel cylindrical nanorods.** $R$, the radius of the rods including the thickness of coated CTAB bilayer; $r$, the inter-rod distance between two parallel cylindrical nanorods; $\sigma$, the effective diameter of the CTAB micelles; $P_0$, the osmotic pressure. Shadowed area represents the overlap volume between two cylindrical nanorods.

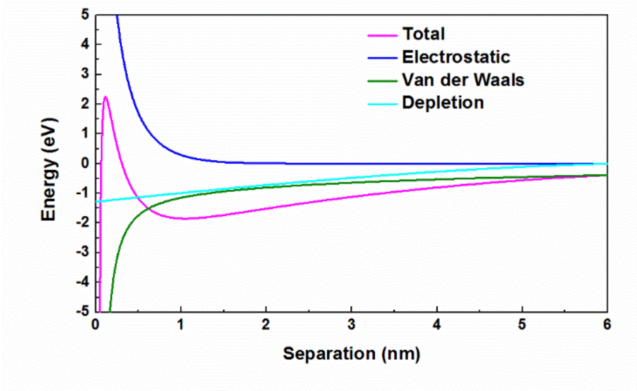


**Supplementary Figure 6. Potential energy for the side-by-side Au nanorod dimer.**

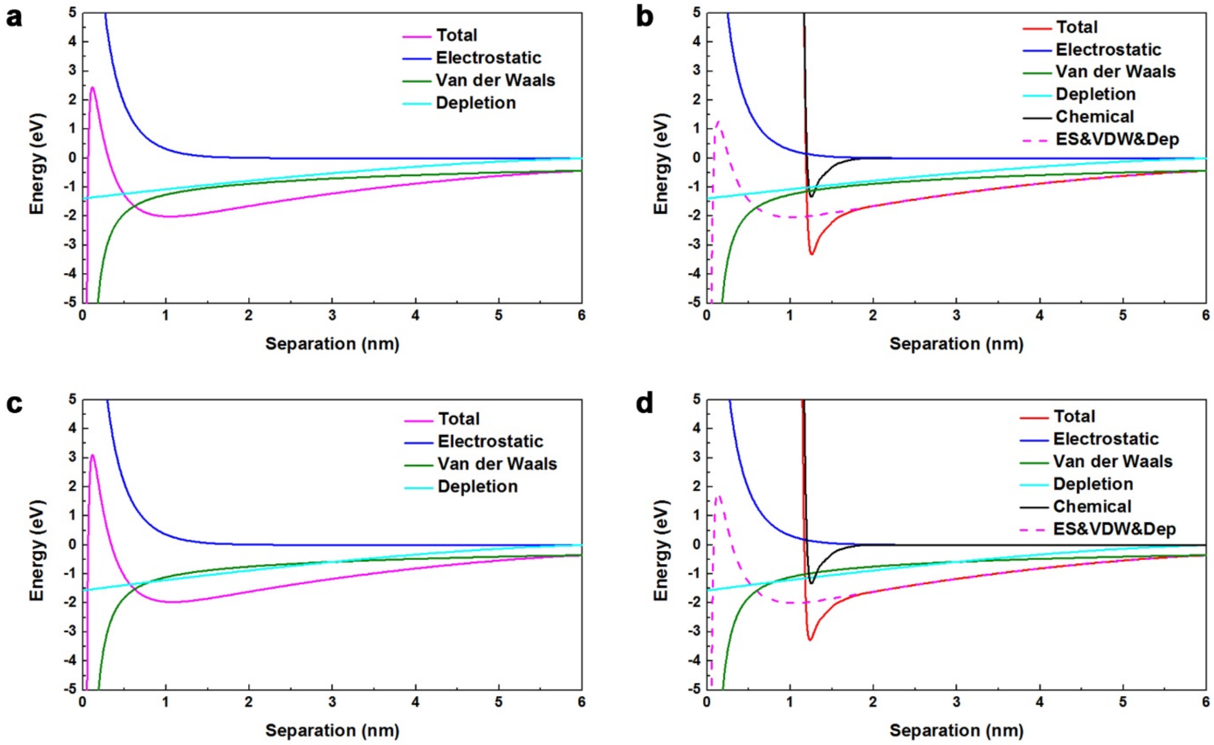


**Supplementary Figure 7. Potential energy for the side-by-side nanorod dimers.** (**a**) Ag dimer without R6G. (**b**) Ag dimer with R6G. (**c**) Pd dimer without R6G. (**d**) Pd dimer with R6G.

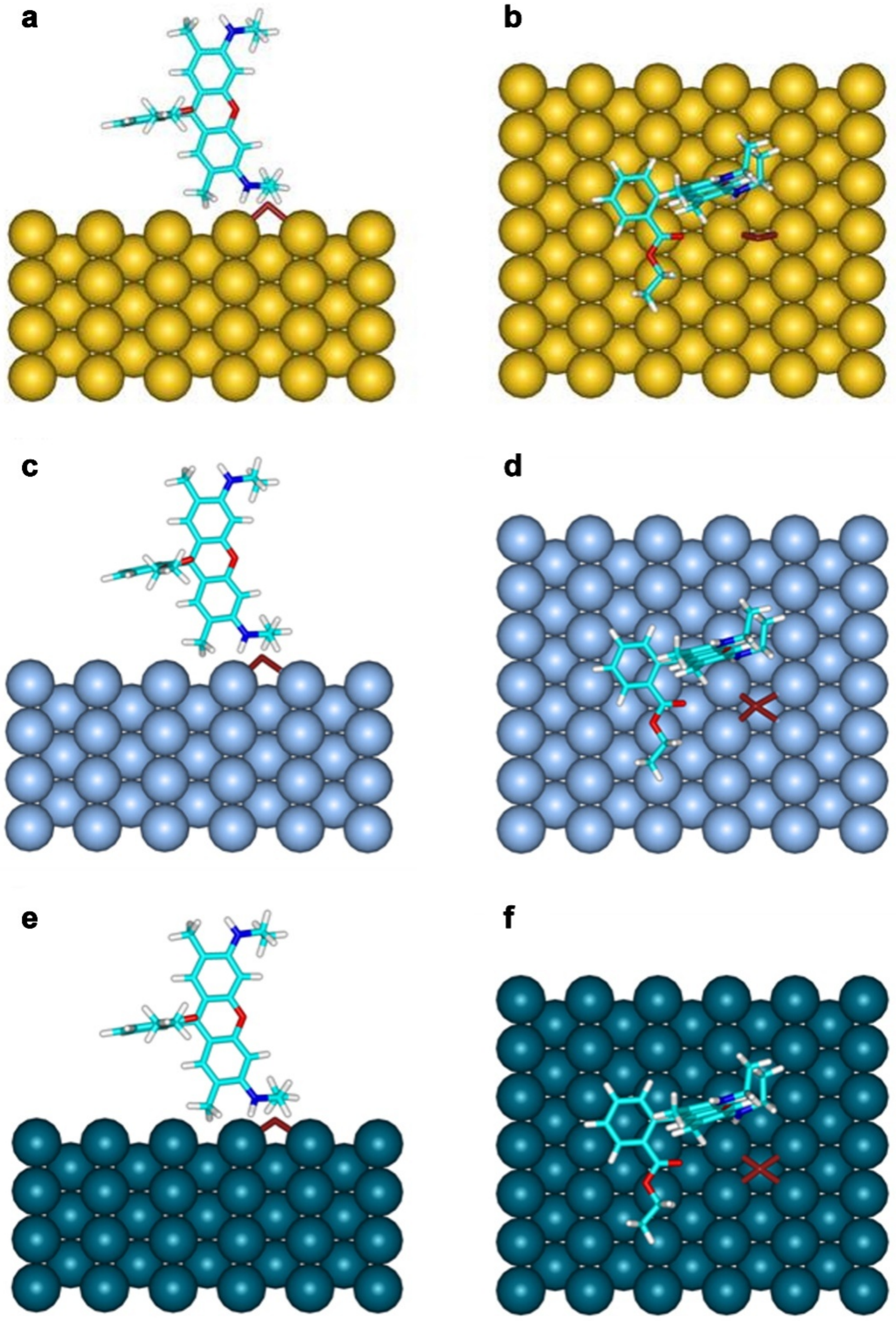


**Supplementary Figure 8. Optimized structure for R6G adsorbed on {110} facets by DFT method.** (**a,b**) Side view and top view on Au. (**c,d**) Side view and top view on Ag. (**e,f**) Side view and top view on Pd. Color scheme: C, cyan; N, violet; O, red; H, grey; Au, golden; Ag, silver; Pd, mazarine; Br, brown.

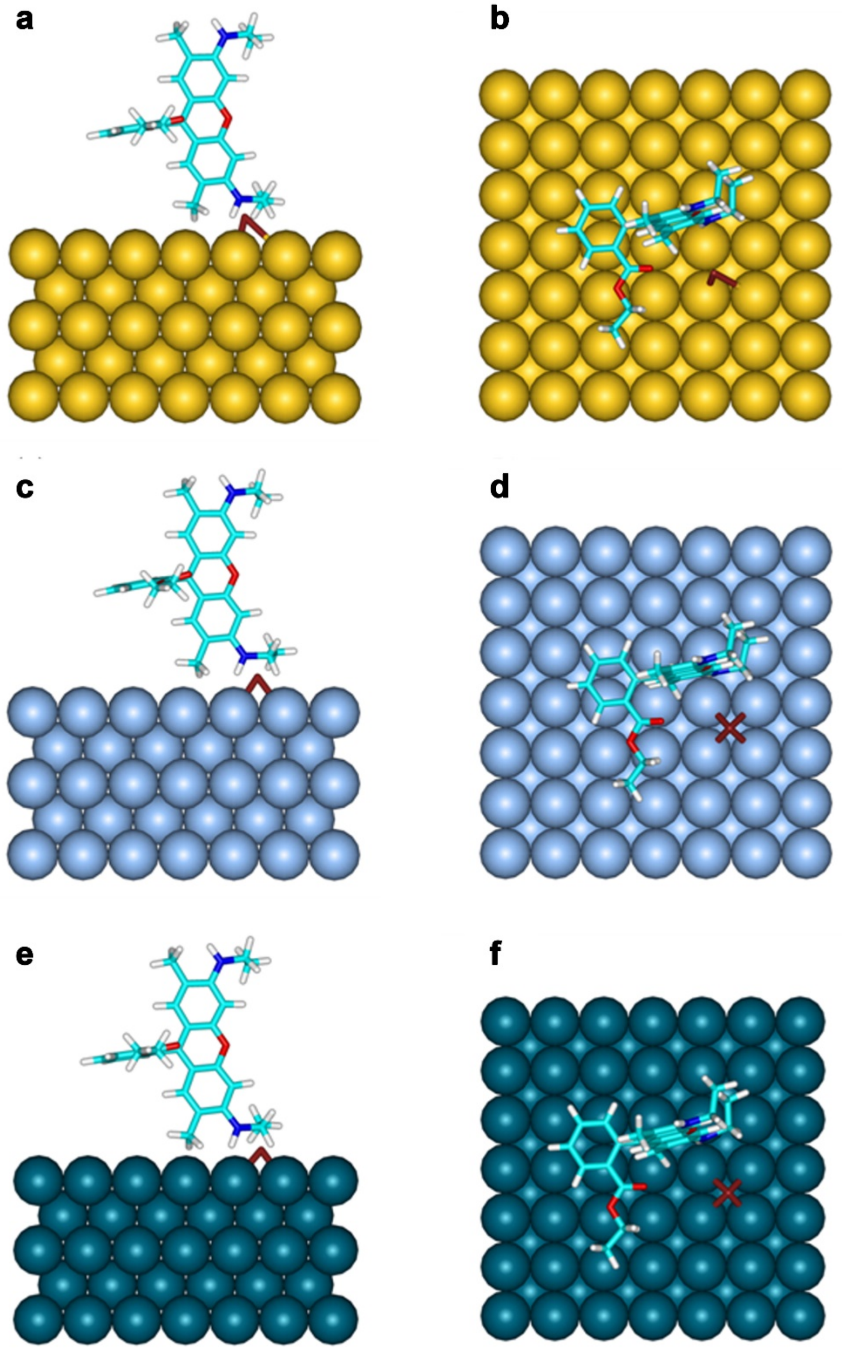


**Supplementary Figure 9. Optimized structure for R6G adsorbed on {100} facets by DFT method.** (**a,b**) Side view and top view on Au. (**c,d**) Side view and top view on Ag. (**e,f**) Side view and top view on Pd. Color scheme: C, cyan; N, violet; O, red; H, grey; Au, golden; Ag, silver; Pd, mazarine; Br, brown.

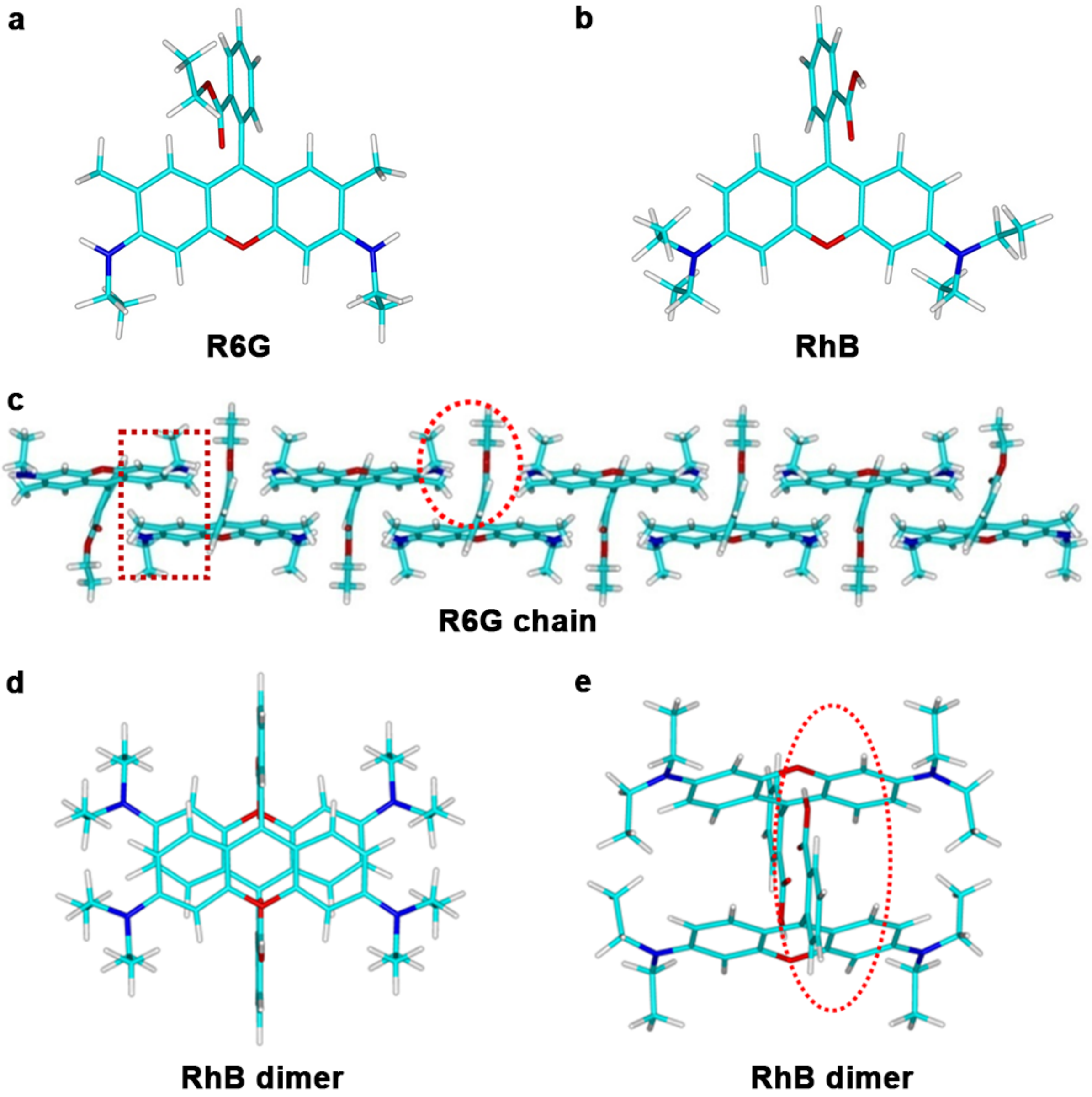


**Supplementary Figure 10. Optimized structures by DFT method.** (**a**) R6G. (**b**) Rhodamine B (RhB). (**c**) R6G chain. The red dashed rectangle (π-π stacking), the red dashed circle (hydrogen bonding). (**d**) RhB dimer (side view). (**e**) RhB dimer (top view). The red dashed circle (hydrogen bonding). Color scheme: C, cyan; N, violet; O, red; H, grey.

R6G molecules prefer to form a rod-like chain in a head-to-tail fashion via π-π stacking and hydrogen bonding. Such R6G J-aggregates on particle surface is observed experimentally[9, 10].

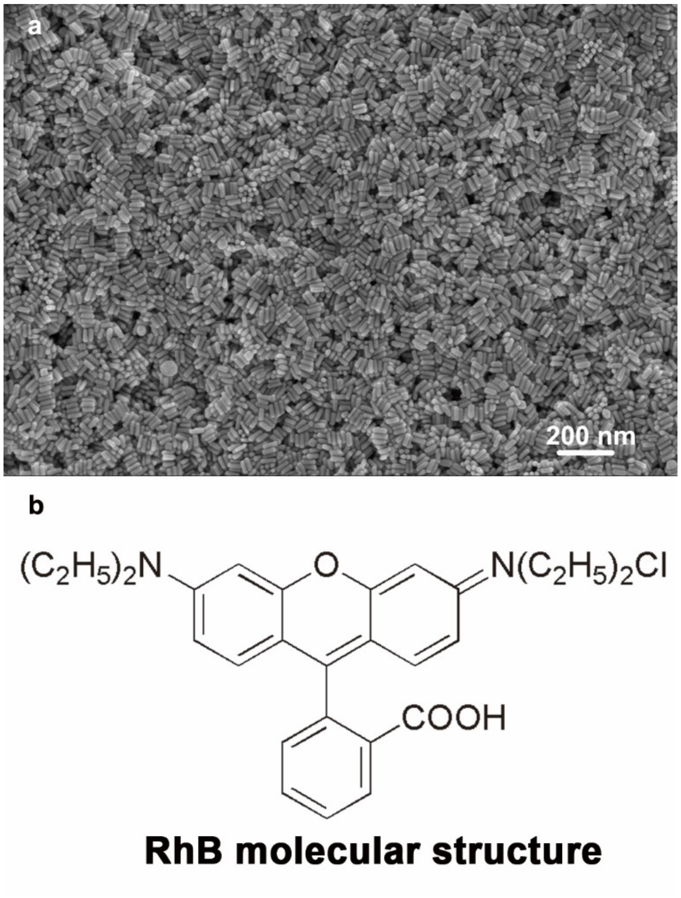


**Supplementary Figure 11. Random rod aggregation by introduction of RhB.** (**a**) SEM image of random rod aggregates. (**b**) RhB molecule structure.

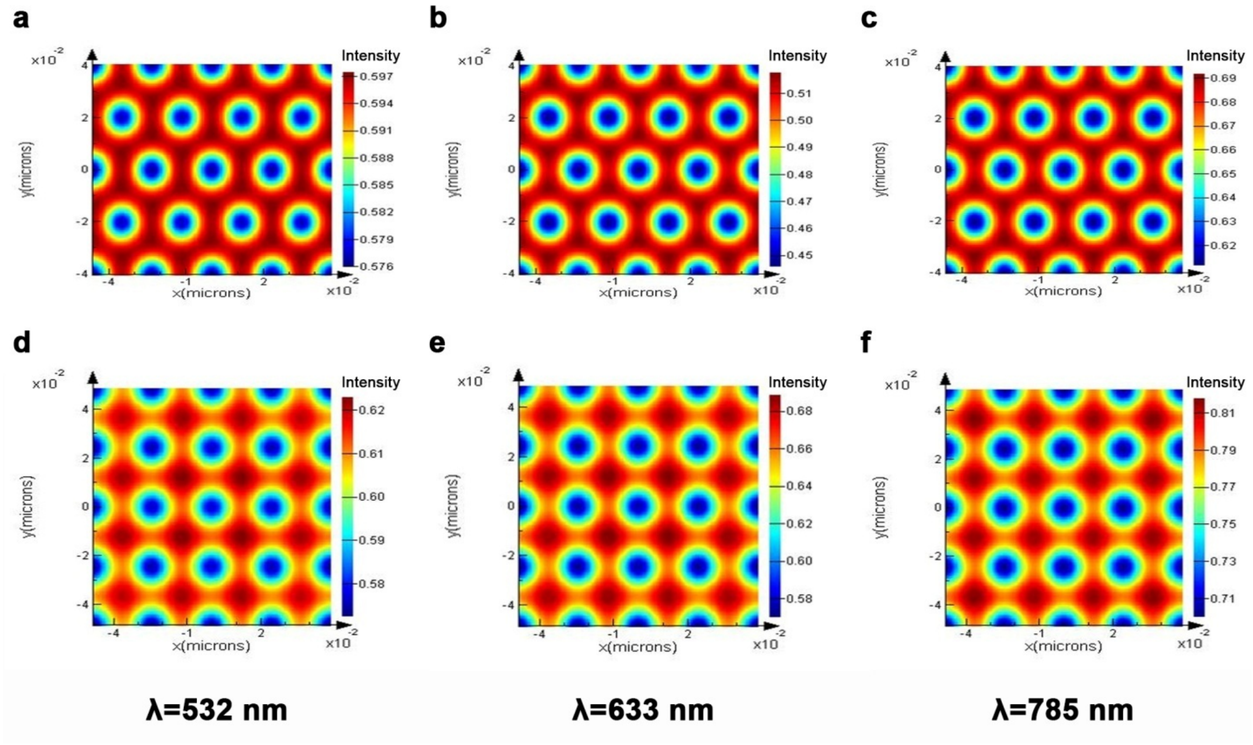


**Supplementary Figure 12. Field intensities of Au nanorod assemblies (*a* = *b* = 21.4 nm) under illumination of three wavelengths.** (**a-c**) 532 nm, 633 nm, 785 nm in hexagonal superlattice, respectively. (**d-f**) 532 nm, 633 nm, 785 nm in tetragonal superlattice, respectively.

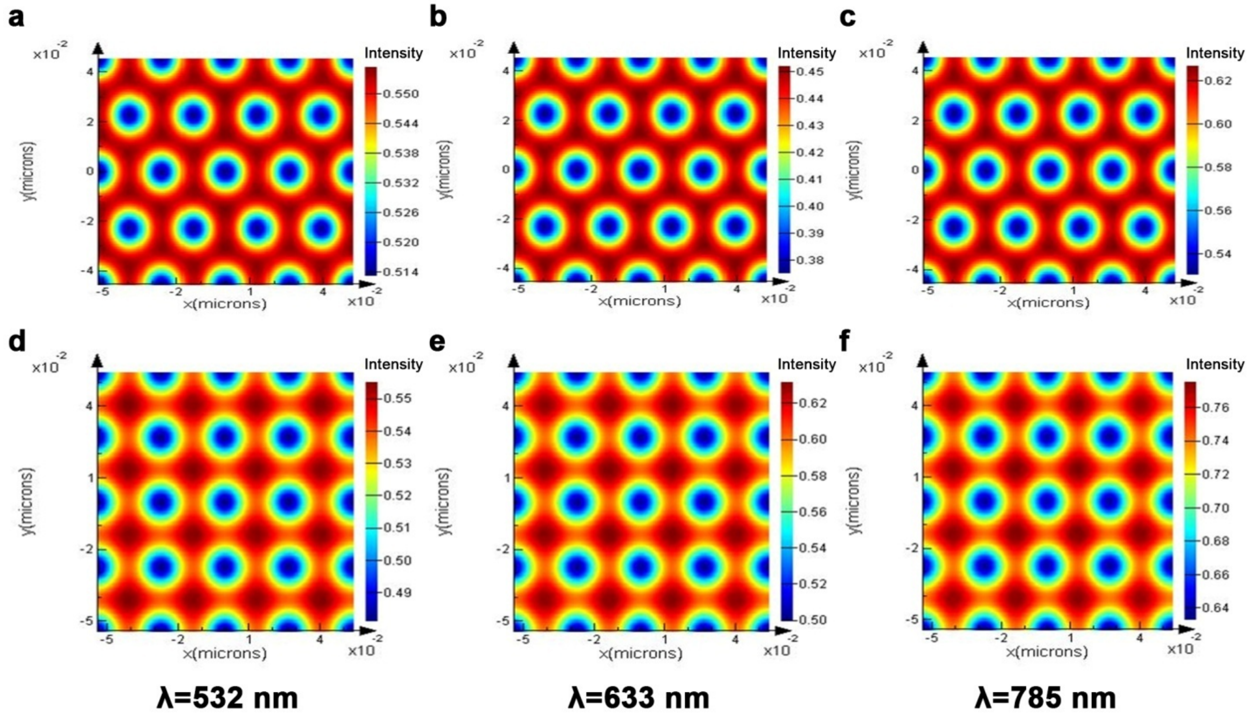


**Supplementary Figure 13. Field intensities of Ag nanorod assemblies (*a* = *b* = 26.4 nm) under illumination of three wavelengths.** (**a-c**) 532 nm, 633 nm, 785 nm in hexagonal superlattice, respectively. (**d-f**) 532 nm, 633 nm, 785 nm in tetragonal superlattice, respectively.

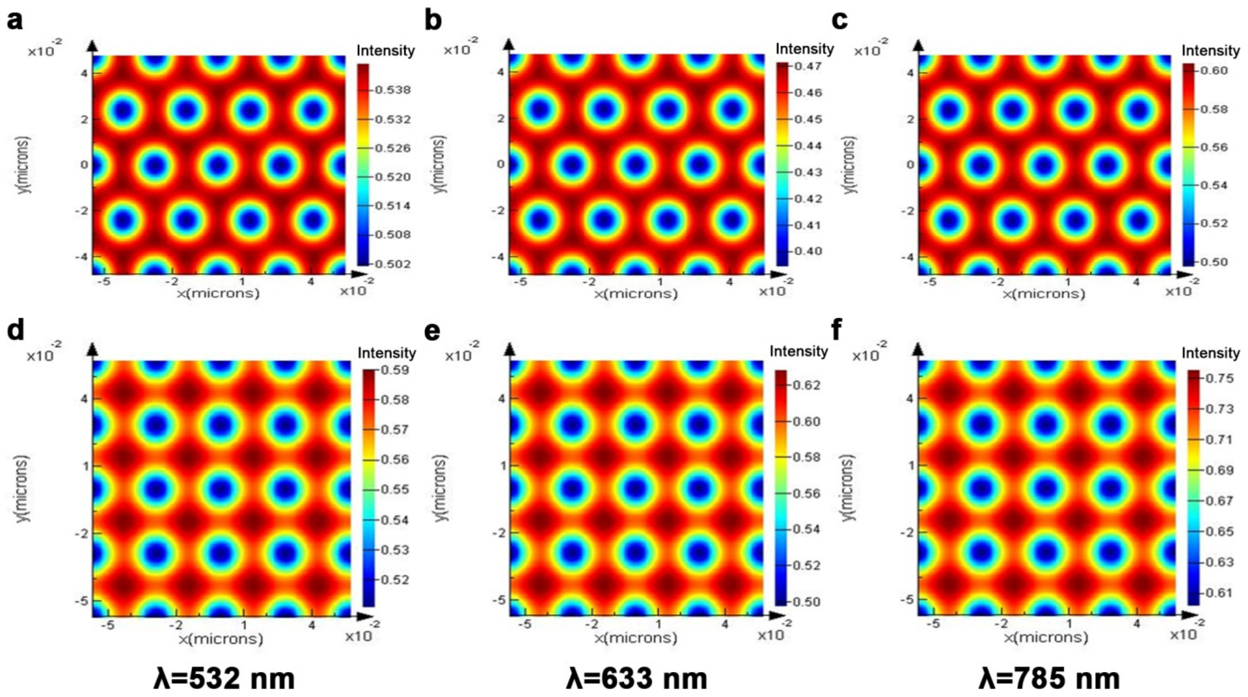


**Supplementary Figure 14. Field intensities of Pd nanorod assemblies (*a* = *b* = 28.4 nm) under illumination of three wavelengths.** (**a-c**) 532 nm, 633 nm, 785 nm in hexagonal superlattice, respectively. (**d-f**) 532 nm, 633 nm, 785 nm in tetragonal superlattice, respectively.

**Supplementary Table**

**Supplementary Table 1. Calculated adsorption energy for R6G on {110}/{100} facets by DFT method.** Here, the more positive energy indicates that the calculated system is more stable.

| | Au{110} | Au{100} | Ag{110} | Ag{100} | Pd{110} | Pd{100} |
|---|---|---|---|---|---|---|
| R6G (eV) | 2.452 | 2.419 | 1.585 | 1.604 | 2.650 | 2.683 |

## Supplementary References


1. Williams, D. B., Carter, C. B. *The Transmission Electron Microscopy*. Springer (2009).
2. Ohshima, H., Hyono, A. Electrostatic interaction between two cylindrical soft particles. *J. Colloid Interf. Sci.* **333**, 202-208 (2009).
3. Russel, W. B., Saville, D. A., Schowalter, W. R. *Colloidal dispersions*. Cambridge University Press (1999).
4. Asakura, S., Oosawa, F. On Interaction between Two Bodies Immersed in a Solution of Macromolecules. *J. Chem. Phys.* **22**, 1255-1256 (1954).
5. Becke, A. D. A multicenter numerical integration scheme for polyatomic molecules. *J. Chem. Phys.* **88**, 2547-2553 (1988).
6. Delley, B. From molecules to solids with the DMol3 approach. *J. Chem. Phys.* **113**, 7756-7764 (2000).
7. Delley, B. An all□electron numerical method for solving the local density functional for polyatomic molecules. *J. Chem. Phys.* **92**, 508-517 (1990).
8. Perdew, J. P., Wang, Y. Accurate and simple analytic representation of the electron-gas correlation energy. *Phys. Rev. B* **45**, 13244-13249 (1992).
9. Sanchez-Valencia, J. R., Toudert, J., Gonzalez-Garcia, L., Gonzalez-Elipe, A. R., Barranco, A. Excitation transfer mechanism along the visible to the Near-IR in rhodamine J-heteroaggregates. *Chem. Commun.* **46**, 4372-4374 (2010).
10. Sanchez-Valencia, J. R., Aparicio, F. J., Espinos, J. P., Gonzalez-Elipe, A. R., Barranco, A. Rhodamine 6G and 800 J-heteroaggregates with enhanced acceptor luminescence (HEAL) adsorbed in transparent SiO2 GLAD thin films. *Phys. Chem. Chem. Phys.* **13**, 7071-7082 (2011).
